# From adaptive licensing to adaptive pathways: Delivering a flexible life‐span approach to bring new drugs to patients

**DOI:** 10.1002/cpt.59

**Published:** 2015-02-04

**Authors:** H‐G Eichler, LG Baird, R Barker, B Bloechl‐Daum, F Børlum‐Kristensen, J Brown, R Chua, S Del Signore, U Dugan, J Ferguson, S Garner, W Goettsch, J Haigh, P Honig, A Hoos, P Huckle, T Kondo, Y Le Cam, H Leufkens, R Lim, C Longson, M Lumpkin, J Maraganore, B O'Rourke, K Oye, E Pezalla, F Pignatti, J Raine, G Rasi, T Salmonson, D Samaha, S Schneeweiss, PD Siviero, M Skinner, JR Teagarden, T Tominaga, MR Trusheim, S Tunis, TF Unger, S Vamvakas, G Hirsch

**Affiliations:** ^1^ European Medicines Agency (EMA) London UK; ^2^ Center for Biomedical Innovation Massachusetts Institute of Technology (MIT) Cambridge MA USA; ^3^ Centre for the Advancement of Sustainable Medical Innovation (CASMI) London UK; ^4^ Medical University of Vienna Vienna AT; ^5^ European network for health technology assessment (EUnetHTA) Danish Health and Medicines Authority Copenhagen DK; ^6^ Pilgrim Health Care Institute and Harvard Medical School Boston MA USA; ^7^ Health Sciences Authority (HSA) Singapore; ^8^ Sanofi‐Aventis, Chilly‐Mazarin FR; ^9^ Global Clinical Research Bristol‐Myers Squibb Wallingford CT USA; ^10^ Novartis Vaccines & Diagnostics ‐ Cambridge MA USA; ^11^ National Institute for Health and Care Excellence (NICE) London UK; ^12^ Zorginstituut Nederland Diemen NL; ^13^ Amgen Ltd Uxbridge UK; ^14^ Collegeville PA USA; ^15^ M4P (Medicines 4 Patients) Consulting London UK; ^16^ GlaxoSmithKline Research Triangle Park NC USA; ^17^ Pharmaceuticals and Medical Devices Agency (PMDA) Tokyo Japan; ^18^ European Organisation for Rare Diseases (EURORDIS) Paris France; ^19^ College ter Beoordeling van Geneesmiddelen Medicines Evaluation Board (CBG/MEB) & University of Utrecht Utrecht NL; ^20^ Health Canada Ottawa ON Canada; ^21^ Bill and Melinda Gates Foundation Seattle WA USA; ^22^ Alnylam Pharmaceuticals, Cambridge, MA, USA and Biotechnology Industry Association (BIO) Washington DC USA; ^23^ Canadian Agency for Drugs and Technologies in Health (CADTH) Ottawa ON Canada; ^24^ Massachusetts Institute of Technology (MIT) Cambridge MA USA; ^25^ AETNA Hartford CT USA; ^26^ Medicines and Healthcare Products Regulatory Agency (MHRA) London UK; ^27^ University of Rome Tor Vergata; ^28^ Läkemedelsverket Uppsala SE; ^29^ Institut national d'excellence en santé et en services sociaux (INESSS) Quebec Canada; ^30^ Harvard Medical School Boston MA USA; ^31^ Agenzia Italiana del Farmaco (AIFA) & Medicine Evaluation Committee (MEDEV) Rome Italy; ^32^ Panel member of the Patient‐Centered Outcomes Research Institute (PCORI) Advisory Panel on Rare Disease Washington DC USA; ^33^ National Organization for Rare Disorders (NORD) Danbury CT USA; ^34^ Sloan School of Management Massachusetts Institute of Technology (MIT) Cambridge MA USA; ^35^ Center for Medical Technology Policy (CMTP) Baltimore MD USA; ^36^ Naia Pharmaceuticals and Massachusetts Institute of Technology (MIT) Cambridge MA USA

## Abstract

The concept of adaptive licensing (AL) has met with considerable interest. Yet some remain skeptical about its feasibility. Others argue that the focus and name of AL should be broadened. Against this background of ongoing debate, we examine the environmental changes that will likely make adaptive pathways the preferred approach in the future. The key drivers include: growing patient demand for timely access to promising therapies, emerging science leading to fragmentation of treatment populations, rising payer influence on product accessibility, and pressure on pharma/investors to ensure sustainability of drug development. We also discuss a number of environmental changes that will enable an adaptive paradigm. A life‐span approach to bringing innovation to patients is expected to help address the perceived access vs. evidence trade‐off, help de‐risk drug development, and lead to better outcomes for patients.

In the 1980s, human immunodeficiency virus (HIV) advocacy groups threw into sharp relief the “evidence versus access” conundrum faced by drug regulators. The conundrum refers to the delicate trade‐offs between encouraging rapid patient access to promising therapies on the one hand and ensuring that patients, and their regulatory and physician proxies, possess adequate information on benefits and harms at the time of marketing authorization on the other.^1^ Similarly, payers, and sometimes patients, must balance uncertainties about the net benefits with the uncertainties about both financial costs and foregone alternative treatment opportunities.

Legislators and drug regulatory agencies have responded to the challenge by introducing flexible licensing pathways. These include accelerated approval (in the US) and conditional marketing authorization/approval (in the EU and Japan) as well as other regulatory tools^2^ for situations where “the benefits to public health of [immediate availability] outweigh the risks inherent in the fact that additional data are still required.”^3^ Payers have responded with managed entry agreements (MEAs), coverage with evidence development (CED), and similar approaches to flexibly develop needed real‐world effectiveness and value information.^4^

These measures are helpful and the flexibility of decision makers has been exercised in the face of recent high‐profile infectious disease outbreaks.^5^ Yet, stakeholders argue that a further evolution and, where possible, alignment of the regulatory and the reimbursement (or payer/coverage) pathways for innovative medicines is needed. There is much debate under the headings of adaptive licensing (AL), medicine's adaptive pathways to patients (MAPPs), staggered approval, progressive authorization, or life‐span approach to licensing and reimbursement. While the headings may be different, the underlying concepts proposed are similar.

In a 2012 publication coordinated by the multi‐stakeholder NEWDIGS collaboration hosted by the MIT Center for Biomedical Innovation, we defined the concept of AL as follows^6^: “*Adaptive licensing is a prospectively planned, flexible approach to regulation of drugs and biologics. Through iterative phases of evidence gathering to reduce uncertainties followed by regulatory evaluation and license adaptation, AL seeks to maximize the positive impact of new drugs on public health by balancing timely access for patients with the need to assess and to provide adequate evolving information on benefits and harms so that better‐informed patient‐care decisions can be made*.”

While the concept was entitled “adaptive licensing,” it was argued that clinical drug development, licensing, reimbursement/coverage, utilization in clinical practice, and monitoring of treatment outcome should be viewed as a continuum and, to the extent possible, should be planned in a prospective and integrated way, with cooperation and input from all stakeholders.^6^

Under AL, the development program is restructured to allow for early approval *and coverage* of a new compound for a limited population, typically (but not necessarily) with a high unmet medical need, based often on smaller initial clinical studies. Approved indications, coverage, and therapeutic value would be revisited at several points along the clinical development pathway as treatment populations are broadened or restricted based on new efficacy and safety data.^6^, ^7^

The 2012 paper on AL^6^ has met with considerable interest. However, some discussants report that AL is a difficult concept to convey to stakeholders. Others, e.g., health technology assessment (HTA) organizations, voice a concern over what they consider the unjustified abandonment of tried and tested pathways to market.^8^ Representatives from healthcare payers may be wary of new early access schemes for often premium‐priced drugs in light of tight budgets. Still others argue that existing systems already allow for broad flexibility which has been used when merited by extraordinary circumstance.

Against this background of ongoing debate and building on the 2012 publication, we aim to clarify some of the concepts of AL that have frequently given rise to debate within the scientific community. We hope this may facilitate the implementation of the AL concept.

We examine the changes in the scientific and political environment that we believe will make AL the preferred approach in the near future (we call these external influences “drivers of AL”; summarized in **Table** 1). We also discuss environmental changes that will enable but not in themselves necessitate a transition from the traditional regulatory and coverage decision framework (“enablers of AL”; **Table** 1).

**Table 1:** Drivers and enablers of adaptive licensing (adaptive pathways)

**Drivers**
Patient expectations: demand for timely access and emphasis on unmet medical need
Emerging science: fragmentation of treatment populations and early disease interception
Healthcare systems under pressure: rise of payer influence
Pharma/investors under pressure: sustainability of drug development
**Enablers**
Improved understanding of disease processes, better knowledge management
Innovative clinical trial designs
Rapid learning systems in the healthcare environment
Bringing patients to the table: understanding acceptable uncertainty
From prediction to monitoring
Targeted prescribing

Since 2012, the concept has evolved and some stakeholders considered that “adaptive licensing” was too narrow a term because the emphasis should be not just on regulation but must include all steps to access, including postregulatory decision making and appropriate use in clinical practice. The term “medicines adaptive pathways to patients” (MAPPs, or “adaptive pathways”) was proposed as a more inclusive concept.^9^ We agree that a broader outlook is needed and shall use both terms as appropriate in this article.

Moving from a conventional to an AL approach requires a range of practical changes and transitions that are summarized in **Table** 2 and discussed below. All of the transitions are predicated on earlier and broader stakeholder involvement as well as transparency to ensure public acceptance of licensing or coverage decisions taken.

**Table 2:** Transitions that are required to move from a conventional scenario to an adaptive licensing (adaptive pathways) scenario

Conventional scenario	Adaptive licensing scenario
**Single gated licensing decision** The life span of a technology is clearly divided into a pre‐ and a post licensing phase by the moment of marketing authorization.^6^	**Life span management** AL acknowledges that knowledge continues to accumulate after a license is granted and that access is best addressed by repeat cycles of “learning‐confirming‐(re)licensing.” Early engagement of decision makers enables integrated planning of drug development, licensing, reimbursement (coverage), utilization in clinical practice, and monitoring of treatment outcome. The life‐span management is expected to lead to lower *realized* risks for patients compared to the current approach—in spite of smaller data packages early on.
**Prediction** Historically, once a drug was authorized, regulators had limited power to monitor performance or influence real‐life use of the drug. This was a responsible justification for demanding high evidence standards in order to predict a drug's performance in the market place. Analogous considerations applied to coverage decisions.	**Monitoring** Regulators in several jurisdictions have been granted substantial new authorities in postlicensing surveillance and risk mitigation; the tools for monitoring real‐world performance (e.g., registries, e‐medical records, postauthorization efficacy studies, methodology to address confounding) are improving, effectively providing a basis for a life‐span approach to marketing authorization. Analogous considerations apply to coverage decisions.
**RCT only** In many therapeutic areas, information from RCTs is almost exclusively the basis for regulatory decisions; information from nonrandomized studies is often not considered robust enough by regulators and sometimes by payers (exceptions may be orphan medicines and postlicensing safety studies).	**Toolkit for evidence generation** The entire toolbox of knowledge generation is used to underpin regulatory and coverage decisions, including conventional RCTs, real‐world (pragmatic) RCTs, and all variations of (nonrandomized) observational studies. Real‐world evidence gains importance to inform postinitial rounds of licensing and coverage. Key is identifying prospectively situations where non‐RCT studies can be convincing.
**Broad populations** Sponsors often aim to obtain as broad as possible an initial license. Effects in identifiable subgroups that are nested within the broad population may (if at all) be addressed subsequently, often for purposes of differentiation against incoming competitor products.	**Targeted populations** An adaptive approach would initially aim to show positive benefit–risk and added value in a defined subpopulation, followed by additional clinical trials and studies in other subpopulations that would lead to gradual widening (or restricting) of the label and the covered populations, as supported by new data.
**Focus on licensing** Obtaining a marketing authorization is the primary goal of sponsors, considerations of (payer) access follow later.	**Focus on patient access** The information needs of all decision makers (including regulators, payers, providers, and patients) are considered from the start and, where possible, are aligned to enable efficient drug development and timely access; patients are increasingly involved in decision making.
**Open utilization** Physicians have near‐complete freedom of prescribing drugs off‐label, without evidence generation.	**Targeted utilization** Greater emphasis by regulators, payers, and industry on targeted drug utilization in the marketplace and on mitigating off‐label use; with a view to ensure safe use, continued learning, and cost‐effectiveness.

AL: adaptive licensing; RCT: randomized controlled trial.

## DRIVERS OF AL

### Patient expectations: demand for timely access and emphasis on unmet medical need

In the words of one patient representative, “the safest drug that no one can afford or that arrives too late is of no benefit to a patient” (HTAi policy forum 2014, Washington, DC). This powerful statement expresses what we consider a key driver for adaptive pathways: growing pressure for timely access by ever more patients and their advocates, at a time of ever more constrained healthcare budgets. Our assessment is based on the evolution of patient advocacy over the past decades. In the 1980s, the call for rapid access to new treatments was heard almost exclusively from HIV advocacy groups. The movement then broadened to include cancer survivors and patient or parent groups from a range of orphan conditions.^10^ More recently, it expanded to advocacy for chronic inflammatory and neurologic conditions, and other chronic diseases.^11^, ^12^ Patient groups are increasingly better informed, better organized, and, in some instances, willing to fund and steer clinical research, as shown by advocacy groups for cystic fibrosis and other conditions.

These patients and their advocates emphasize that drug development and market access should not only benefit patients in some distant future state but should also address the unmet needs of the current generation of patients.

**Figure 1:**
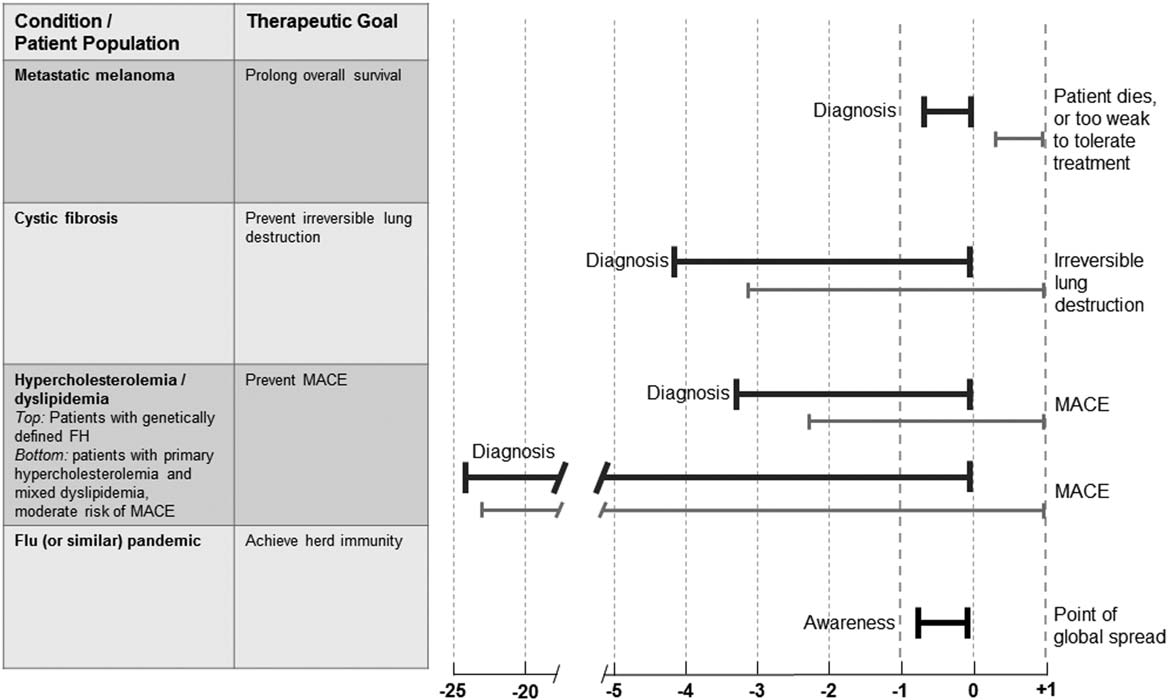
Treatment window of opportunity. The schematic illustrates why the time of access to a promising treatment is relevant to patients with any serious condition, independent of the time course of the disease. Treatment windows of opportunity are shown for a few exemplary serious conditions ranging from very short (metastatic melanoma) to extremely long disease courses (primary hypercholesterolemia and mixed dyslipidemia). Estimates of windows are symbolized by bold horizontal lines, the start‐ and endpoints are described for each condition; the lengths of the lines are for illustration purposes only, and are not based on epidemiological data. Note that the time of the endpoint of the treatment window of opportunity may not be known for many health states (e.g., hypercholesterolemia) and the window period may in reality end long before the time of the events shown in the figure (e.g., because irreversible vascular damage has occurred before the MACE occurs). Nonetheless, all patients will eventually reach the endpoint of their treatment windows of opportunity; this is illustrated by the right‐alignment of the horizontal lines. The thin lines underneath each bold line are intended to show that each year a new cohort of patients with the same condition emerges and will reach the endpoint of their window 1 year after the previous cohort. This is true for all conditions where the year‐on‐year incidences remain relatively stable and in the absence of other emerging treatment options. The dashed vertical lines illustrate that a difference in time to market access of, for example, 2 years (year –1 vs. year +1) will have the same effect on patients with metastatic melanoma as for patients with hypercholesterolemia, insofar as the two annual cohorts of patients who are at the end of their treatment window of opportunity will gain or lose an opportunity to benefit from promising treatment. It follows that the time course of a disease *per se* should not be a driver of the evidence vs. access debate. The obvious but rare exceptions to this rule will be conditions with highly variable incidence rates. This is illustrated by the last example, flu (or similar) pandemic, where urgency of access is primarily determined by the anticipated time to peak global spread. The two different treatment window of opportunity lines under the “hypercholesterolemia/dyslipidemia” heading are intended to show that subgroups of patients within the same broad condition may have very different windows. FH: familial hypercholesterolemia; MACE: major adverse cardiac events.

It should come as no surprise that initial calls for early access came from patient groups with immediately life‐threatening diseases which run their course very quickly, such as untreated HIV or terminal cancers. The urgency of access is intuitive in these cases, and “unmet need” may be perceived as being a function of the dynamics of a disease. Yet we submit that urgency of access and unmet need is not necessarily related to the pace at which disease progresses. To explain the argument, we introduce the concept of “treatment window of opportunity.” We define the treatment window of opportunity as the median period in months or years during which patients with a disease can potentially benefit from a novel treatment. The window is bounded by the date of diagnosis and the date when treatment becomes impossible or futile because the patient dies or because there is another point of no return, like the disease becomes nonresponsive, treatment comes too late to change the disease course, or some irreversible damage has already occurred, such as irreversible joint damage in children with juvenile rheumatoid arthritis. By analogy, the treatment window of opportunity for preventive treatments would begin at the time when a high risk of a disease has been established for an individual patient. **Figure** 1 illustrates the concept based on a number of exemplary conditions and treatment goals. Assuming that the incidence of a given condition (that is the number of new cases per year) remains fairly constant year‐on‐year, it follows that the urgency of access to promising treatments is independent of disease dynamics: for every year without access, the window will shut on one annual cohort of patients, whether the window is short or long (**Figure** 1).

Considering this clinical perspective, it seems entirely appropriate that patients with chronic, slow, irreversibly progressing diseases and for which there are only unsatisfactory treatment options make the same plea for urgency of access as do those with fast progressing conditions. From a patient's perspective, duration of the disease course should not be the key input variable when making the access vs. evidence trade‐off (**Figure** 1).

The bioethical and practical question that is at the heart of the “too late” issue has been framed, in the words of one bioethicist, as whether “future patients will have to accept some degree of less certainty in their treatments for the benefits of current patients.”^13^

How does the adaptive pathways concept seek to recast this ethical dilemma and achieve an appropriate trade‐off between “unmet need” and “less certainty”?

First, AL does not advocate a purely needs‐driven licensing and access policy; such policy would result in ineffective and unsafe treatments coming to market—simply because a clinical condition is desperate and there is no alternative. We concur with other commentators that some proposed needs‐driven approaches that seek to essentially deregulate the introduction of new therapies are not in the interest of patients and would remove incentives to better understand and make drugs ever better.^14^ Under the AL paradigm, the pathways and speed of patient‐access should be driven by the likelihood of a new treatment successfully addressing the unmet need. Hence, the approach considers simultaneously the patients' situation and the potential efficacy and safety of the treatment under study.

In practice, this requires decision makers and companies to be transparent about the decision‐making process and the underlying assessment of both unmet need and the known facts about a new product, as well as be clear about risks and remaining uncertainties.

It is encouraging that some healthcare decision makers are pioneering the use of metrics, such as *shortfall of quality‐adjusted life years* or even a capability approach,^15^ which could help quantify unmet medical need.^16^, ^17^ We may not see in the near future a universally acceptable metric; however, a burden of illness or loss of health concept that takes into account the shortfall of quality of life may be more appropriate than the speed of disease progression to death to guide decision makers in their judgments about the level of acceptable uncertainty.

Likewise, the long‐term promise of a new treatment is difficult to quantify early on but the decision about whether to accept a new treatment on a smaller evidence base can be guided by, for example, exceptional response rates on some likely surrogate endpoint in small patient cohorts or the considerations laid out in the criteria for breakthrough therapy designation by the US FDA.^18^

Second, AL is not about changing the benefit–risk trade‐offs. We recall that risk and uncertainty are very different concepts, although often conflated in public debate.^10^ Under any licensing or coverage paradigm, the expected benefits should outweigh the expected risks for a defined patient population—anything else is unethical. The issue is whether the uncertainties around the benefit and risk estimates must have been resolved to a standard of clear and convincing evidence (in the US usually referred to as “substantial evidence”) at the time of the initial licensing and coverage decisions or whether a positive decision is acceptable on the basis of a well‐reasoned and transparently communicated “balance of probabilities with continuous monitoring.” For most disease conditions, achieving a standard of clear and convincing evidence will take longer and may thereby deny access to current patients—as was originally pointed out by the HIV advocates in the 1980s.

Third, any acceptable “degree of less certainty”^13^ about a specific product can only be temporary, even in the face of high unmet need. A fundamental tenet of AL is progressive reduction of uncertainty by way of preagreed evidence generation plans and time frames. In addition, the AL approach emphasizes tight management of utilization, monitoring in the market place, and an ability and political willingness to restrict use or withdraw a product if the benefit–risk or the value for money is less than expected. Together, these precautions are expected to lead to lower *realized risks* for patients compared to the current approach (see below).

Fourth, a starting point for the AL concept is the growing realization that almost every disease or clinical indication consists of multiple subpopulations. (For an example of substratification of even a classic blockbuster indication like dyslipidemia, please refer to Baird et al^19^; the argument will be further developed below.) Hence, the ethical question about the trade‐off between the interests of future vs. current patients will likely have a different answer for each individual subpopulation. What is acceptable uncertainty for one subgroup may not be acceptable for another, and will be dependent on the patient subgroups' disease burden, potential for benefit, and, to the extent practical, on patients' declared preferences to accept a given level of uncertainty in exchange for access to new therapies.

### Emerging science: fragmentation of treatment populations and early disease interception

An additional driver towards AL is the growing fragmentation of treatment‐eligible populations. The profound impact on therapeutic outcomes of ever more predictive stratification criteria is illustrated by the almost century‐long evolution of hematologic malignancies: starting from the ill‐defined concept of “cancer of the blood,” an increasingly better understanding of pathologies led to a growing number of defined subpopulations with hematologic malignancies. This knowledge has, in turn, enabled the testing and routine use of tailored treatment regimens which greatly improved patient well‐being and survival.

The past decade has seen a raft of binary disease stratifications, based on (genotypic) biomarkers and dedicated companion diagnostic. An often‐quoted example for efficacy stratification is cetuximab (Erbitux; MerckSerono, Lilly, Bristol‐Myers Squibb) which is active only in certain tumors with KRAS wildtype but had little or no effect in tumors harboring a KRAS mutation.^20^ Abacavir (Ziagen; GlaxoSmithKline) shows what stratification can do for drug safety: the detection of a genetic marker enabled prescribers to preidentify patients who are at high risk of developing a hypersensitivity reaction to abacavir. Screening out those likely to develop a hypersensitivity reaction allows for the majority of patients to continue to benefit from the drug.^21^ This is remarkable because, in the past, often a majority of patients was denied the potential benefits of a treatment to protect a few patients who might experience a serious adverse event but could not be identified beforehand.

Most diseases have a genetic component that explains only part of the response to a drug (e.g., cetuximab) and will define a (higher) probability of response but no guarantee. Yet binary disease stratification helped improve the benefit–risk and cost‐effectiveness of many novel drugs. It required regulators and other decision makers to address issues around the codevelopment of the test‐drug pair as well as questions of clinical trial design. Yet these were only adaptations of the existing paradigms for evidence generation, licensing, and coverage decisions.

It seems reasonable to predict that, within the next decade, we will see substratification being taken to a more sophisticated level that will fundamentally challenge contemporary regulatory and coverage decision‐making.

In part, this will result from the emergence of personalized treatment combinations based not on one but multiple binary stratifications. For example, multiple tumor‐driving mutations have been found in some malignancies.^22^ Administering a panel of biomarkers to detect multiple genetic aberrations (or other combinations of predictive markers) and multistratified trials will be required to translate this knowledge into therapeutic strategies. This development is now starting in oncology and may expand to other therapeutic areas. It holds promise to improve treatment efficacy but has rightly been described as “terra incognita” for drug development programs, regulatory approval pathways,^23^ and coverage decisions. Even the most innovative of current clinical trial designs, like “basket” or “umbrella” trials, are not designed to address this level of complexity.^24^ Benefit–risk or added value judgments for many patient subgroups will no longer be supported by conventional, adequately powered randomized controlled trials (RCTs) but will necessarily be associated with uncertainties that can only be addressed over the longer term.

A different kind of multistratified medicine is illustrated by ivacaftor (Kalydeco, Vertex Pharmaceuticals), a drug that engages the cystic fibrosis transmembrane regulator (CFTR), a gene product for which more than 1,900 different mutations are currently known (although not all of them are necessarily disease‐causing). Interaction of the CFTR‐directed drug molecule with the various target structures corresponding to the many mutations may be heterogeneous. This heterogeneity, in turn, results in a large number of potential benefit–risk strata or, from a healthcare payer's perspective, a large number of different value propositions. Some mutations are more common than others and RCTs will be feasible in only a few subgroups. Hence, the level of evidence will be quite different across mutation groups and uncertainty will need to be progressively reduced over time. This is reflected in the evidence development plan agreed between (European) regulators and the sponsor, which foresees conventional RCTs, crossover studies, or uncontrolled case series for individual patient subgroups.^25^ It is conceivable that benefit–risk information for some of the very rare mutations can only be accrued quite late in the product's life span and based on real‐world data.

We consider ivacaftor to be an example of the adaptive pathway to market that personalized treatments will travel in the future; in many healthcare environments, this will include repeat adjustments of the treatment‐eligible population for which the treatment is reimbursed.

Lastly, the appearance of growing numbers of “custom‐made” medicines can be anticipated. With some classes of therapeutics, such as antisense oligonucleotides, preparations of modified patient‐derived cells, bacteriophages, or other types of advanced therapies, each individual patient would receive their own individualized treatment. Hence, the treatment‐eligible population for a given preparation is an “*n* of 1” and the benefit–risk profile may or may not be similar across the patient‐treatment pairs; both on‐target and off‐target activities may differ. Johnston and Feldschreiber have described the challenges to the traditional model of phase I, II, and III trials and to current pharmaceutical legislation that arise from development of antisense oligonucleotides.^26^ They call for an essentially adaptive development and licensing approach based on a “master antisense oligonucleotide product” (which will obtain its license through a conventional development program) and “custom‐made products” which will be developed and authorized along different pathways.^26^ This approach may provide even less comfort in extrapolating the safety and efficacy to the smaller subsets than there is with the ivacaftor example, because minor changes in the molecular structure of a drug can result in a significant change in the toxicity profile. Yet it may be the only viable route to market.

To conclude, the progressive fragmentation of clinical indications and treatment populations will necessitate the revamping of many of the conventional paradigms for clinical trials, regulatory evidence requirements, and for coverage decisions in several ways.

Adequately powered RCTs, the standard for drug approvals and coverage decisions, may not be feasible in a growing number of subpopulations because only few patients fit within a given disease stratum.

On the other hand, RCTs can become smaller as trial populations are enriched for potential responders and anticipated effect size increases. This is welcome, as it reduces the time and cost of conducting trials. However, the ability to detect rare adverse events is a function of the number of patients observed. As the total number of trial patients diminishes because efficacy is shown earlier, the knowledge base about safety is smaller at the time of initial market authorization and coverage. Adaptive pathways anticipate progressive learning about safety within a predefined and monitored patient usage group. Adaptive pathways can therefore help develop the needed safety data for those patients for whom the safety uncertainty has been accepted without initially exposing larger numbers of other patients to the product.

Uncertainty around benefit and risks will fluctuate across subpopulations. Subpopulations will sometimes be identified on the basis of post‐hoc and nonmultiplicity adjusted subgroup analyses and benefit–risk assessment will rely on a “weight of evidence approach,” including biological plausibility, relationships of subgroups to overall population, and preclinical data showing unique or enhanced drug effects on populations of interest. Extrapolation, often by way of modeling and simulation, from one patient subpopulation to the next will become more common in cases where the biology of the disease and the drug's mode of action are well understood.

For many subpopulations, the life span approach to licensing and coverage and learning from real‐world experience as advocated by adaptive pathways will become the only viable access route to new treatments.

The access vs. evidence question will also be magnified as a result of emerging opportunities to intercept chronic, slowly progressing diseases very early on. Consider a likely scenario for future research into Alzheimer's disease (AD): whatever the appropriate target for delaying disease progression may turn out to be (the “amyloid hypothesis” or some amyloid‐independent mechanisms^27^), there is a growing body of data suggesting that any successful intervention may need to start perhaps 15–20 years before manifestation of clinical symptoms.^28^ It will be nearly impossible to demonstrate an effect of early disease interception on patient‐relevant endpoints by way of conventional RCTs. Running a double‐blind RCT over such a time period to await the emergence of significant intergroup differences in clinical outcomes is impractical. It may also be difficult to convince sponsors to take the risk of committing significant funds to large trials over such long periods.^29^ It follows that promising drugs for early disease interception will have to be licensed and covered on the basis of surrogate endpoints^30^ that are reasonably expected to predict clinical benefit but are only validated retrospectively—it would take as long to prospectively validate the endpoints as to do the RCTs.

Some commentators may consider all this to lead to an unacceptable lowering of evidence standards but the alternative is to ignore the progress of medical science and risk foregoing the potential benefits of early disease intercepting treatments. We submit that adaptive pathways are the only viable approach to these situations and that regulators, payers, and society at large will have to become more sanguine with levels of uncertainty over an initial time period. However, acceptance of uncertainty must be counterbalanced by a realistic, transparent, and preagreed pathway for continued evidence generation. It also requires regulatory authority for early withdrawal if residual risk tips the benefit–risk balance and willingness across all stakeholders including payers, patients, and providers to stop the use of these treatments if therapeutic value cannot ultimately be confirmed.

### Healthcare systems under pressure: rise of payer influence

A very different force that is starting to motivate a shift in policies towards adaptive pathways comes directly from the healthcare environment. Across the world, only a small and shrinking fraction of new, high‐priced drug treatments are fully paid out‐of‐pocket by patients. It follows that the decisions made by third‐party payers whether to reimburse, or how to tier copayments, gain increasing importance to both patients and marketing authorization holders. Regulatory approval has become merely a necessary but no longer sufficient precondition for patient access.

Some payers, or HTA bodies that advise them, currently emphasize that the “full” information package about a drug's performance has to be available at the time of the first coverage decision.^31^ However, there is growing awareness among many other payers that they, like the regulators, cannot escape the access vs. evidence conundrum. In fact, public debate about reimbursement tends to be even more acrimonious than about licensing because the financial element is absent from the regulatory decision making. Payers are coming to recognize that the binary concept of *experimental* vs. *medically necessary* is based on a simplified view of evidence and uncertainty—and that more nuanced policy mechanisms are necessary to align with the continuous nature of strength of evidence. A growing number of payers therefore move from conceiving HTA and reimbursement as a one‐off snapshot, to seeing them as ongoing processes aiming at providing greater certainty about value for money as evidence accumulates.^4^

Moreover, payers have for some time realized that the only reliable way to effectively manage costs in the long term is by providing treatments in more efficient ways. This entails better targeting of the treatment‐eligible population with the goal of reducing the number‐needed‐to‐treat (NNT)—that is, the number of patients that need to be treated (and reimbursed) in order to achieve one desired therapeutic outcome. A high NNT raises cost for healthcare payers without generating value. It is not surprising that emerging effectiveness guidelines which seek to better inform payers' coverage decisions call for more granular subgroup information.^32^

Once the coverage decision has been made, payers need to take a keen interest in ensuring appropriate prescribing, a high level of patient adherence, and real‐time monitoring of treatment outcomes in order to realize the anticipated value for money. These pressures on healthcare payers converge to making an adaptive pathway to market a necessity for them.

On the operational level, the paradigm shift is becoming apparent by the growing number of managed entry agreements (MEAs) concluded in some healthcare environments, although uptake of these arrangements has not been uniform across payers, especially in the US. MEAs are voluntary formal arrangements between payers and manufacturers with the aim of sharing the financial risk due to uncertainty around the clinical and cost‐effectiveness of new technologies at the time of introduction.

MEAs can take different forms, including performance‐based agreements, coverage with evidence development (CED), and disease management programs. For an overview and SWOT analysis of MEA/CED, please refer to Ferrario and Kanavos.^33^

MEAs are likely here to stay. However, while conceptually straightforward, implementation of MEAs can be challenging in practice. In order to realize their full potential, these arrangements need to have the flexibility to allow new information on a drug's performance to trigger price changes in either direction and to change the scope of the covered population. There are currently still few precedents for agile payer mechanisms to modify price or covered populations.

The flexibility of MEAs in addressing postinitial licensing uncertainty and enabling access to expensive treatments provides an opportunity for synergies with regulatory initiatives. Under an AL paradigm we anticipate a growing number of postauthorization safety and/or efficacy studies imposed by regulators. There is no compelling reason why these studies could not be prospectively planned and aligned with postlicensing evidence generation foreseen by payers under an MEA/CED scheme—provided that a “learning healthcare system” is in place (discussed below).

AL might help get greater acceptance of MEAs in the US because AL will naturally establish clear criteria for MEAs, and payers can reduce exposure to uses outside the AL approvals, which has become a key concern.

A recent analysis of coverage decisions in the EU showed that a sizable fraction of compounds approved under conditional marketing authorization (CMA) was subsequently reimbursed with a MEA. (CMA is an EU regulatory pathway similar to “Accelerated Approval” in the US and, in spirit, close to the AL concept, although narrower in scope.) The MEAs put in place comprised initial restriction of reimbursement for small high unmet‐need subpopulations, performance, or financial risk‐sharing and ongoing evidence development plans.^34^

While these actions are not (yet) coordinated between the EU regulators and payers, they lay the groundwork for more coordination of the overall market access pathway. In the EU, formal dialogs are now starting between the European Medicines Agency (EMA) and a number of HTA bodies (for instance, by the framework of EUnetHTA) to explore consensus mechanisms for continual evidence generation. In the US, the FDA and the Medicare program have experimented with a parallel review process as well as CED programs that have involved ongoing dialog between the agencies, device companies, professional societies, and other stakeholders—demonstrating the feasibility of this approach.^35^

### Pharma/investors under pressure: sustainability of drug development

The low productivity of biopharmaceutical research and development (R&D) undermines the ability of the industry to address the growing needs of healthcare across the world. To a large extent, the productivity gap is the result of factors external to the topic of this article.^36^

However, conventional development and licensing pathways seem economically inefficient, at least for common chronic indications such as neurodegenerative and cardiovascular diseases, where sponsors are forced to lock themselves into large long‐term outcome trials that are essentially big bets with low probability of success.^36^ The unintended consequence is that it is driving consolidation, with fewer companies and fewer late‐stage development programs in these conditions as well as lower total output to address medical need.

It has been postulated that “redesigning clinical trials to include fewer patients, providing conditional approval of drugs, and requiring postmarketing surveillance could have a profound effect” on overall development costs. The paradigm‐shift “would lower the threshold for financing a drug's development so that more drugs would be brought forward.”^37^ While these assumptions have not all been put to the test, at least one analysis of past industry performance suggests that development programs targeting smaller, better‐defined populations have higher overall success rates than those aiming at larger, heterogeneous populations.^36^

**Figure 2:**
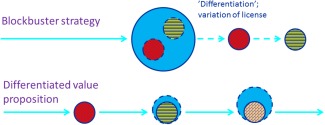
The transition from “big‐to‐small” towards “small‐to‐big” as proposed under an adaptive pathway. Under the blockbuster strategy, sponsors would initially aim to obtain a license and coverage population that is as broad as possible (symbolized by the large blue circle). Effects in identifiable patient subgroups that are nested within the broad population (symbolized by small colored circles) would be addressed subsequently, often for purposes of differentiation against incoming competitor products. By contrast, an adaptive approach would initially aim to show positive benefit–risk and added value in a defined subpopulation, followed by additional clinical trials and studies in other subpopulations. A potential beneficial effect of this staggered approach is use of more targeted extrapolation (where justified) and nontraditional (e.g., observational) studies which in turn may reduce the total number of patients required to enroll in interventional clinical trials. The total treatment‐eligible population will grow in sequential steps over time (symbolized by the light blue circles).

These considerations, as well as the move towards personalized medicine and the need for better described value propositions for payers, accelerate the transition from the blockbuster to the niche buster business model for drug developers^38^; in other words, to transition from “big‐to‐small” towards “small‐to‐big,” as illustrated in **Figure** 2.

Combine this with other elements of the adaptive pathways concept (**Table** 2), and the risks of a given development program can be staggered, leading to, perhaps, a reduction in overall risk.

Adaptive pathways offer a unique opportunity to address another growing burden for the innovation ecosystem: over time, individual payers or HTA organizations have increased their evidence requirements. While regulators have achieved some degree of interregional harmonization of evidence standards, HTA bodies are at an earlier point in that dialog.^39^ The lack of alignment results in different technical, scientific, and economic hurdles for drugs to be developed. The emphasis of the adaptive pathways approach on early planning with engagement from all invested stakeholders, including patients, is expected to catalyze important consensus building between different payers/HTA bodies within and perhaps across regions.

Acknowledging these challenges, a growing number of pharma executives are supporting the adaptive pathways approach, and the European Federation of Pharmaceutical Industries and Associations (EFPIA) is now calling for the creation of “the framework required to successfully implement ‘Medicines Adaptive Pathways for Patients’ (MAPPs).”^9^

## ENABLERS OF AL

We postulate that the environmental changes described above converge to make adaptive pathways a necessity in future for the majority of new drug products.

At the same time, a set of legal, logistic, and scientific preconditions have been identified that need to be met, both in the “front end” (prior to initial licensure) and in the “back end” (after initial licensure) for AL to be successful. We here summarize a number of recent developments that will go a long way to enable the implementation of adaptive pathways (see **Table** 1).

### Improved understanding of disease processes, better knowledge management

In‐depth knowledge of the natural history of diseases, existing baseline data on, for example, symptom severity, treatment adherence rates, as well as other epidemiology aspects gleaned from existing databases or emerging large data networks^40^ and reanalysis of past trials^41^ helps to make RCTs more efficient and identify surrogate endpoints, and may increasingly obviate the need for concurrent control groups, e.g., in rare diseases. This knowledge and data can also be leveraged for the postinitial licensing evidence generation foreseen under AL, by providing a reference point against which the real‐world performance of a treatment can be assessed.^6^

### Innovative clinical trial designs

Adaptive trials offer an opportunity to assess accumulating results at given timepoints with the possibility of modifying the trial design: for example, by changing the trial population to focus on patient subsets that are responding better to the experimental therapy or—in the context of AL—when a license has been granted for one subset to another near‐label population. Adaptive trials afford a key opportunity to meet the information needs of both regulators and payers in the same trials^42^ and operational continuity from pre‐ to postauthorization phases.^43^

Adaptive trial designs can also help defuse the tension arising in some instances between generation of robust evidence (which may require a placebo arm in an RCT) and access to promising therapies in clinical trials by minimizing placebo exposure of patients through interim adjustments.

More efforts are required to refine the concept of adaptive trial designs but considerable progress has been made over the past years. Regulators in the EU and US have provided broad guidance,^44^, ^45^ and various types of adaptive trials with different goals have been successfully implemented.^46^, ^47^

### Rapid learning systems in the healthcare environment

Even with these advances in clinical trial designs, RCTs will always leave significant uncertainty about benefits, risks, and real‐life utilization and performance of new drugs; RCTs are often designed to remove confounding factors such as comorbidities or exclude elderly, frail patients. “Confounder cleansing” increases the ability to detect a drug effect if it is there, but reduces external validity. Progressive reduction of those uncertainties will need to be achieved by way of the use of data from observational studies. We emphasize that observational studies should not replace but complement RCTs. In the eyes of some methodologists, nonrandomized studies have for a long time taken a back seat for evidence generation—if not worse. Indeed, some observational studies have later been found to be misleading.^48^

Capabilities within three key domains are important to make observational studies a valuable source of information: data and infrastructure, methodology to address the inherent limitations of nonrandomized information, and, lastly, operational enablers including, for example, organizational processes, mindsets, and legal frameworks. On all frontiers, great strides have been made over the past years to enable learning in the postinitial licensing period.^49^

In many jurisdictions, the quantity of electronic data in health records or dedicated registries has expanded rapidly. These data are becoming increasingly standardized, reliable, and complete. Some patient reported outcomes, treatment adherence data, morbidity, and activities of daily living outcomes are likely to become routine in future as e‐health records expand and data compatibility is increased.^50^

At the same time, methodologies have been proposed and developed to address, to the extent possible, the issue of confounding and other biases when drawing conclusions based on observational studies.^51^, ^52^ Lastly, processes have been put in place to enable collaboration of data owners through common data models, common protocols to query the datasets, and governance models.^53^

These welcome developments have enabled regulators to reach important conclusions about drug safety in the real world. For example, the Mini‐Sentinel Initiative of the FDA has shown how “big data [can be] rendered fit and functional.”^50^ Likewise, the European Network of Centres for Pharmacoepidemiology and Pharmacovigilance (ENCePP), coordinated by the EMA,^54^ and the MIHARI project in Japan^55^ are being leveraged to address drug safety issues prioritized by regulators. Further, the US Patient‐Centered Outcomes Research Institutes is developing “PCORnet,” a large distributed clinical data network to support comparative effectiveness research.^53^

In the US, the National Medication Safety, Outcomes and Adherence Program (NMSOAP) will begin a pilot study to track real‐time responses to a small number of selected medications. Access to patients' electronic medical records and direct contacts with patients are designed to generate a longitudinal database that can answer prespecified or post‐hoc questions.^56^ Focusing on cancer, the American Society of Clinical Oncology (ASCO) is developing CancerLinQ, a knowledge‐generating computer network that will collect and analyze cancer care data from millions of patient visits, together with expert guidelines and other evidence, to generate real‐time, personalized guidance and quality feedback for physicians.^57^

Progress in the generation and utilization of real‐world data is uneven between and within regions. Not all relevant questions can be addressed by real‐world data today (see below) but as the building blocks of observational research continue to be refined and more fully utilized, non‐RCT information will become increasingly relevant for assessment of benefits, risks, comparative effectiveness, and value.

### Bringing patients to the table: understanding acceptable uncertainty

How much uncertainty around benefits and risks can be accepted has always been a key question for healthcare decision makers. Given the inescapable trade‐off between timely access and evidence that AL seeks to address, the question becomes ever more central. We consider it self‐evident that patients' views should be paramount when judging what is acceptable clinical uncertainty for a given treatment scenario, but obtaining representative views from patients is an ongoing mutual learning process for both patients representatives and decision makers.

A growing number of regulatory agencies and HTA bodies are inviting patients to declare their preferences (e.g., about the importance of clinical trial endpoints), value judgments, and benefit–risk trade‐offs.^58^, ^59^ Experience to date shows that patient representatives and advocates do not invariably push for early access, at any cost, but have often expressed their balanced acceptance levels of risks and uncertainty.^10^

It is equally encouraging to see a number of academic studies and pilot projects (e.g., on “patient juries”) starting to address the issue.^60^, ^61^ These initiatives, while still in their infancy, seek to establish methodology to inform decision makers on patient preferences, including the level of uncertainty that patients may be willing (not forced) to accept. Explicit and reproducible input from patients should facilitate the decision of regulators and payers to allow drug access at a given level of uncertainty, by lending legitimacy and public acceptance of the decision. Actively engaging patients in decision making about their own care will enhance transparency and build trust in AL.

Broader and more systematic involvement in decision making of patients and their advocates also offers an opportunity to enlist patient support for the secondary use of health data (or the setting up of registries) to enable evidence generation through the postlicensing phase.

### From prediction to monitoring

Many years ago, a common adage among regulators used to be that once a drug “is out the door” their powers to monitor and steer use and to detect or mitigate risks was limited. This was, at the time, a responsible justification for demanding high preapproval evidentiary requirements in order to address residual uncertainty and predict a drug's performance in the marketplace. An unintended consequence was an increase in the cost of drug development and delay of therapies to reach the market. Yet progress has been made over the past years on two fronts that may enable regulators to move from a prediction to a monitoring paradigm.

First, regulators in several jurisdictions have been granted substantial new authorities and mandated engagement in postlicensing surveillance and risk mitigation, effectively providing a legal basis for a life span approach to market authorization.^62^, ^63^, ^64^ Regulators can now impose and enforce on marketing authorization holders an array of postlicensing requirements to refine benefit–risk information, although the legal basis to do so is different across jurisdictions and is more limited in the US than, e.g., in the EU.

Second, mindsets, awareness, and infrastructures have significantly changed within many healthcare environments. Consider the dramatic evolution of postlicensing identification of adverse drug effects. Thalidomide use in pregnancy can cause phocomelia in babies, an adverse effect that is both highly visible and has a low background incidence. Yet in the 1950/60s it took around 10,000 cases worldwide before healthcare professionals made the connection between exposure (thalidomide use) and outcome (phocomelia). Contrast this tragically slow learning with the rapid detection of signals for exposure‐outcome pairs like natalizumab and progressive multifocal leukoencephalopathy (PML) in 2005 (three cases before the connection was made) or the 2009 H1N1 pandemic flu vaccine Pandemrix where the first investigations started after the Swedish Medicines Agency had received only six reports from healthcare professionals of narcolepsy following vaccination.^65^ While these are anecdotal cases, they highlight the difference between an *inherent* risk (that is, the likelihood of an adverse drug reaction in a given treatment setting, either known or unknown) and a *realized* risk (that is, the absolute number of patients actually harmed). The inherent risk is a given for a particular drug in a defined setting, but the realized risk can—and has been—reduced dramatically through postmarketing surveillance and risk management.

A recent systematic analysis of risk management plans in the EU showed that regulatory postlicensing requirements could resolve a sizable fraction of open questions about the safety profile.^66^

On a more cautionary note, our ability to detect adverse drug reactions that give rise to relatively small risk ratios on high‐background events (such as myocardial infarctions in diabetic patients) or to quantify real‐life effectiveness is still limited. Yet efforts are under way to systematically explore how a combination of patient‐level data from observational cohort studies, routinely collected healthcare databases, and authorization dossiers can help avoid biased results when conducting early postlaunch observational research into desired or undesired treatment outcomes.^67^

Taken together, the evolutions in legal frameworks, science, and the healthcare environment enable regulators to adopt a life‐span approach to learning and risk management, as is envisaged by the AL concept.

There are parallels between the evolutions of the regulators' and the payers' perspectives.

The authority of regulators in the postlicensing space is complemented by the power of payers, which enables them to foster a continuous learning process over the life span of a drug by way of, for example, coverage with evidence development agreements (see above). However, there are concerns from payers that, under an adaptive pathways approach, they may be locked into costly reimbursement schemes that do not generate value for money if early expectations of value are not confirmed later. Hence, for payers to fully embrace the transition from *prediction* to *monitoring* would require that at least one of two conditions is met: either 1) a political willingness to stop reimbursement for all (or subpopulations of) patients if follow‐up data indicate lower than expected value. This may be difficult to achieve politically in some healthcare environments and may not be acceptable for patients. Or 2) alternatively (preagreed) flexible coverage and pricing structures must be in place to reflect changes in demonstrated value.

### Targeted prescribing

Inappropriate prescribing diminishes benefit–risk and the value of treatments under any licensing or coverage paradigm. Yet the gap between efficacy, as initially assessed by regulators and payers, and real‐world effectiveness could be further magnified under AL. We have discussed that AL places much emphasis on subgroup‐specific information because the level of uncertainty that is acceptable for one subgroup may not be acceptable for another.

Hence, when a drug is initially intended for use in only a well‐defined subset of patients, widespread use in nontarget patients might open the door to negative patient outcomes and payer economics.

Regulators have some, albeit limited, tools to steer drug utilization by way of controlled access programs, prescriber restrictions, educational requirements, clinical reminder systems (to remind prescribers of the need for certain clinical screening), or other measures, such as pregnancy prevention programs.

It emerges, however, that in many healthcare environments payers, healthcare systems providers, and professional societies, rather than regulators, are the stewards of appropriate prescribing. As discussed above, the need to ensure value for money leads to the need for increasingly regimented utilization of new, premium‐priced drugs. For an individual product, this can be achieved by measures including preauthorization requirements, prescribing audits, prescriber restrictions, or tiered copayments. On a more systematic level, healthcare payers increasingly manage the use of new medicines in both the primary care and specialty care sectors by mandating treatment protocols.^68^

Most payers are concerned about off‐label use but cannot be expected to manage drug utilization throughout the entire life span; their incentives and means to do so will also likely decline as drugs become available as generics. However, under an AL paradigm, appropriate prescribing is most critical during the early on‐market phase. We predict that various combinations of regulator and payer actions with cooperation from bodies that produce clinical practice guidelines will address the requirement for early market access across healthcare environments and jurisdictions. It is likely to be most easily achieved in diseases treated only in a few specialist centers, suggesting that these may be good places to start.

## CONCLUSION

Environmental changes affect all components of the healthcare ecosystem and challenge the traditional way of bringing pharmaceutical innovation to patients. In combination, these changes are transformative and will require novel approaches to balance the trade‐off between timely access with the need for evidence. The responses to these challenges will differ across healthcare environments but they will most likely lead to life‐span based, adaptive pathways to patient access in one form or another. We discussed how a number of recent developments enable the transitions from a traditional to an adaptive approach as summarized in **Table** 2.

Further important steps towards enabling adaptive pathways are currently being taken. Regulators have just begun to explicitly address and communicate “uncertainty” in their templates for benefit–risk assessment.^69^, ^70^ A growing number of regulators and payer (or HTA) organizations involve patients in their decision‐making processes.

Yet additional challenges remain in both the “front end” and “back end” parts of an adaptive approach.

In some jurisdictions novel legislative tools may be required to ensure the economic viability of AL, e.g., limited data exclusivity duration after the initial license may turn out as a disincentive.

While collaboration between sponsors, regulators, HTA bodies, and payers throughout the product life span has started in the EU, other jurisdictions, notably the US, do not have a national healthcare system where decisions on access/payment are centrally managed. Hence, implementation of this part of adaptive pathways will be more challenging in the US.

AL requires the political will to limit access to an approved drug to a subset of the population, which is not in line with the current prevailing approach in the US of “practice of medicine” to allow for off‐label use. Moreover, experience has shown that it may be politically challenging to remove a drug from the market or restrict payment should the initial benefit–risk balance not be confirmed postapproval.

These issues will require substantial (political) debate among the various stakeholders. In the US, the bipartisan “21st Century Cures initiative” is expected to develop policies and tools which would dovetail with the concept of adaptive pathways.^71^ The feasibility of the adaptive pathways approach is currently being explored in the context of the EMAs pilot project on AL.^72^

While the conceptual change of the adaptive pathways concept is transformative, implementation is expected to be evolutionary rather than disruptive, and will likely progress differently across jurisdictions.

Reflecting on the history of surgery, Schlich^73^ pointed out that, with the introduction of antiseptics in the 19^th^ century, “operations whose performance would have been considered insane or criminal just 15 years earlier were now performed routinely.” The reason for this dramatic progress was that with antiseptics the likelihood of wound disease became amenable to risk management. We consider this a pertinent analogy for the history of bringing new drugs to market. In this article we summarized the transformative environmental changes that will make the life‐span approach to drug research, licensing, and reimbursement (aka, adaptive licensing or adaptive pathways) “an operation performed routinely”—and an operation that will be highly beneficial for both patients and the healthcare ecosystem.

## CONFLICTS OF INTEREST

Richard Barker is a non‐executive director of Celgene and iCo Therapeutics; John Ferguson is a Novartis employee and stockholder; Sarah Garner is an employee of NICE. She receives an IMI grant via employment; Gigi Hirsch is the Executive Director of the MIT Center for Biomedical Innovation (CBI). CBI receives consortium membership fees, research grants, and unrestricted grants from a range of non‐profit organizations as well as corporate sponsors, listed at http://cbi.mit.edu/community/; Anton Hoos is Principal of M4P Consulting and has been a paid consultant to pharmaceutical industry. At time of publication, he will be an employee of Amgen; Paul Huckle is an employee and shareholder of GSK; John Maraganore is an employee, CEO, and director of Alnylam Pharmaceuticals; a director of Agios Pharmaceuticals and Bluebird Bio; a Venture Partner with Third Rock Ventures; and a member of BIO, where he serves on the Executive Committee, Chair of the Emerging Company Section, and Co‐Chair of the Regulatory Environment Committee (REC); Sebastian Schneeweiss is consultant to WHISCON, LLC and to Aetion, Inc., a software manufacturer of which he also owns shares. He is principal investigator of investigator‐initiated grants to the Brigham and Women's Hospital from Novartis, and Boehringer Ingelheim unrelated to the topic of this study; Mark Trusheim is President of Co‐Bio Consulting, LLC which serves clients in the life sciences industries.

## SUPPORTING INFORMATION

Additional Supporting Information may be found in the online version of this article.

## Supporting information

Supporting Information

## References

[1] Woodcock, J. Evidence vs. access: can twenty‐first‐century drug regulation refine the tradeoffs? Clin. Pharmacol. Ther. 91:378–380 (2012). 22343812 10.1038/clpt.2011.337

[2] Baird, L. *et al* Accelerated access to innovative medicines for patients in need. Clin. Pharmacol. Ther. 96:559–571 (2014). 25006877 10.1038/clpt.2014.145

[3] Commission Regulation (EC) No 507/2006. <http://ec.europa.eu/health/files/eudralex/vol‐1/reg_2006_507/reg_2006_507_en.pdf>. Accessed 4 July 2014.

[4] Henshall, C. & Schuller, T. HTAi Policy Forum. Health technology assessment, value‐based decision making, and innovation. Int. J. Technol. Assess. Health Care. 29:353–359 (2013). 23845404 10.1017/S0266462313000378

[5] Tominaga, T. , Ando, Y. , Nagai, N. , Sato, J. & Kondo, T. Balancing societal needs and regulatory certainty: the case study of peramivir in Japan. Clin. Pharmacol. Ther. 93:342–344 (2013). 23422872 10.1038/clpt.2012.268

[6] Eichler, H.G. *et al* Adaptive licensing: taking the next step in the evolution of drug approval. Clin. Pharmacol. Ther. 91:426–437 (2012). 22336591 10.1038/clpt.2011.345

[7] Woosley, R.L. & Rice, G. A new system for moving drugs to market. Issues Sci. Technol. (Winter), 63–39 (2005).

[8] Husereau, D. , Henshall, C. & Jivraj, J. Adaptive approaches to licensing, health technology assessment, and introduction of drugs and devices. Int. J. Technol. Assess. Health Care. 12:1–9 (2014). 10.1017/S0266462314000191 24921416

[9] EFPIA: The right prevention and treatment for the right patient at the right time. Strategic Research Agenda for Innovative Medicines Initiative 2. <http://www.efpia.eu/documents/101/61/Strategic‐Research‐Agenda‐for‐Innovative‐Medicines‐Initiative‐2>. Accessed 3 July 2014.

[10] Eichler, H.G. *et al* The risks of risk aversion in drug regulation. Nat. Rev. Drug Discov. 12:907–916 (2013). 24232377 10.1038/nrd4129

[11] Durham, M.S. Tysabri approved for funding: background. <http://www.msdurham.com/treatments/tysabri‐approved‐for‐funding‐background> (2010). Accessed 8 July 2014.

[12] Roth, D. A third seat at the table: an insider's perspective on patient representatives. Hastings Center Report, Volume 41, Number 1, January‐February 2011 p 29–31. 10.1002/j.1552-146x.2011.tb00097.x 21329103

[13] Keane, M. A free‐market approach to clinical data gathering is more ethical. Am. J. Bioeth. 13:19–21 (2013). 10.1080/15265161.2013.814732 23952825

[14] Bianco, P. & Sipp, D. Sell help not hope. Nature. 510:336–337 (2014). 24955467 10.1038/510336a

[15] Coast, J. Strategies for the economic evaluation of end‐of‐life care: making a case for the capability approach. Expert Rev. Pharmacoecon. Outcomes Res. 14:473–482 (2013). 10.1586/14737167.2014.914436 24784902

[16] NICE . NICE seeks views on how it assesses drugs and other technologies. <http://www.nice.org.uk/News/Press‐and‐Media/nice‐seeks‐views‐on‐how‐it‐assesses‐drugs‐and‐other‐technologies‐for‐the‐nhs> (2014). Accessed 3 July 2014.

[17] KCE Drug reimbursement systems: international comparison and policy recommendations. KCE reports 147C. <https://kce.fgov.be/sites/default/files/page_documents/KCE_147C_Drug_reimbursement_systems_4.pdf> (2010). Accessed 3 July 2014.

[18] FDA . Guidance for industry. Expedited programs for serious conditions—drugs and biologics. <http://www.fda.gov/downloads/Drugs/GuidanceComplianceRegulatoryInformation/Guidances/UCM358301.pdf> (2014). Accessed 3 July 2014.

[19] Baird, L. , Teagarden, R. , Unger, T. & Hirsch, G. New medicines eight years faster to patients: blazing a new trail in drug development with adaptive licensing. Scrip Regulatory Affairs 2013 May 24.

[20] Lièvre, A. *et al* KRAS mutation status is predictive of response to cetuximab therapy in colorectal cancer. Cancer Res. 66:3992–3995 (2006). 16618717 10.1158/0008-5472.CAN-06-0191

[21] European Medicines Agency . EPAR Ziagen. European Medicines Agency. <http://www.ema.europa.eu/docs/en_GB/document_library/EPAR_‐_Product_Information/human/000252/WC500050343.pdf> (Last updated 18 Apr 2011).

[22] Sleijfer, S. , Bogaerts, J. & Siu, L.L. Designing transformative clinical trials in the cancer genome era. J. Clin. Oncol. 31:1834–1841 (2013). 23589555 10.1200/JCO.2012.45.3639

[23] Willyard, C. ‘Basket studies’ will hold intricate data for cancer drug approvals. Nat. Med. 19:655 (2013). 23744135 10.1038/nm0613-655

[24] Institute of Medicine . Implementing a National Cancer Clinical Trials System for the 21st Century: Second Workshop Summary. Washington, DC: National Academies Press; 2013 p 36. 24872986

[25] EMA . European Medicines Agency decision. EMA/113589/2014. <http://www.ema.europa.eu/docs/en_GB/document_library/PIP_decision/WC500165454.pdf> (2014). Accessed 3 July 2014.

[26] Johnston, J.D. & Feldschreiber, P. Proposal for new European pharmaceutical legislation to permit access to custom‐made anti‐sense oligonucleotide medicinal products. Br. J. Clin. Pharmacol. 77:939–946 (2014). 24750439 10.1111/bcp.12250 PMC4093919

[27] Pimplikar, S.W. , Nixon, R.A. , Robakis, N.K. , Shen, J. & Tsai, L.H. Amyloid‐independent mechanisms in Alzheimer's disease pathogenesis. J. Neurosci. 30:14946–14954 (2010). 21068297 10.1523/JNEUROSCI.4305-10.2010 PMC3426835

[28] Pike, K.E. *et al* Beta‐amyloid imaging and memory in non‐demented individuals: evidence for preclinical Alzheimer's disease. Brain. 130:2837–2844 (2007). 17928318 10.1093/brain/awm238

[29] Burke, M. Why Alzheimer's drugs keep failing. <http://www.scientificamerican.com/article/why‐alzheimer‐s‐drugs‐keep‐failing/>. Accessed 18 July 2014.

[30] Kozauer, N. & Katz, R. Regulatory innovation and drug development for early‐stage Alzheimer's disease. N. Engl. J. Med. 368:1169–1171 (2013). 23484795 10.1056/NEJMp1302513

[31] Kenny, M. A Q and A with Thomas Müller. <http://www.rajpharma.com/home/news/A‐QandA‐with‐Thomas‐Muller‐adaptive‐licensing‐sceptic‐from‐Germanys‐health‐technology‐assessment‐body‐the‐G‐BA‐337982>. Scrip Regulatory Affair 2012 Dec 7.

[32] CMTP: CMTP issues recommendations for late phase drug studies in type 2 diabetes. <http://www.cmtpnet.org/news‐room/view/cmtp‐issues‐recommendations‐for‐late‐phase‐drug‐studies‐in‐type‐2‐diabetes/> (2014). Accessed 3 July 2014.

[33] Ferrario, A. & Kanavos, P. Managed entry agreements for pharmaceuticals: the European experience. <http://ec.europa.eu/enterprise/sectors/healthcare/files/docs/mea_report_en.pdf> (2013). Accessed 3 July 2014.

[34] Spearpoint, P.A. , Yip, C.Y. & Zhang, W. Lessons for adaptive licensing: analysis of conditionally approved EMA compounds, their reimbursement status and regulatory/reimbursement data requirements. Value Health. 17:A100 (2014).

[35] CMS Coverage Decision Based on FDA Parallel Review Program. <http://www.raps.org/regulatoryDetail.aspx?id=6692>.

[36] Hay, M. , Thomas, D.W. , Craighead, J.L. , Economides, C. & Rosenthal, J. Clinical development success rates for investigational drugs. Nat. Biotechnol. 32:40–51 (2014). 24406927 10.1038/nbt.2786

[37] Kocher, R. & Roberts, B. The calculus of cures. N. Engl. J. Med. 370:1473–1475 (2014). 24571723 10.1056/NEJMp1400868

[38] Trusheim, M.R. , Berndt, E.R. & Douglas, F.L. Stratified medicine: strategic and economic implications of combining drugs and clinical biomarkers. Nat. Rev. Drug Discov. 6:287–293 (2007). 17380152 10.1038/nrd2251

[39] Kristensen, F.B. *et al* Practical tools and methods for health technology assessment in Europe: structures, methodologies, and tools developed by the European network for Health Technology Assessment, EUnetHTA. Int. J. Technol. Assess. Health Care. 25(Suppl 2):1–8 (2009). 10.1017/S0266462309990626 20030885

[40] The National Patient‐Centered Clinical Research Network . <http://www.pcornet.org/>. Accessed 31 July 2014.

[41] Rabinowitz, J. , Werbeloff, N. , Stauffer, V. , Mandel, F. & Kapur, S. More efficient drug discovery trials in schizophrenia: insights from 29 placebo‐controlled randomised controlled trials (RCTs) from the NEWMEDS repository. Presented at the 24th Congress of the European College of Neuropsychopharmacology, Paris, September 3–7, 2011 <http://conferenceservices.elsevier.nl/11ecnp/index.cfm?fuseaction=CIS2002&hoofdnav=Search&content=zk.results_all&topicselected=*&searchtext=rabinowitz&what=FREE%20TEXT&selection=ALL&abstrnbr=S.11.03>.

[42] Eichler, H.G. *et al* Relative efficacy of drugs: an emerging issue between regulatory agencies and third‐party payers. Nat. Rev. Drug Discov. 9:277–291 (2010). 20186141 10.1038/nrd3079

[43] Selker, H.P. *et al* A proposal for integrated efficacy‐to‐effectiveness (E2E) clinical trials. Clin. Pharmacol. Ther. 95:147–153 (2014). 24060819 10.1038/clpt.2013.177 PMC3904553

[44] FDA . Guidance for Industry Adaptive Design Clinical Trials for Drugs and Biologics. <http://www.fda.gov/downloads/drugs/guidancecomplianceregulatoryinformation/guidances/ucm201790.pdf> (2010). Accessed 3 July 2014.

[45] EMA . Reflection paper on methodological issues in confirmatory clinical trials planned with an adaptive design. <http://www.ema.europa.eu/docs/en_GB/document_library/Scientific_guideline/2009/09/WC500003616.pdf> (2007). Accessed 3 July 2014.

[46] Kim, E.S. *et al* The BATTLE Trial: personalizing therapy for lung cancer. Cancer Discov. 1:44–53 (2011). 22586319 10.1158/2159-8274.CD-10-0010 PMC4211116

[47] Tabernero, J. *et al* VE‐BASKET, a Simon 2‐stage adaptive design, phase II, histology‐independent study in nonmelanoma solid tumors harboring BRAF V600 mutations (V600m): activity of vemurafenib (VEM) with or without cetuximab (CTX) in colorectal cancer (CRC). J. Clin. Oncol. 32:5s, 2014 (suppl; abstr 3518). <http://meetinglibrary.asco.org/content/132653‐144> (2014). Accessed 31 July 2014.

[48] Young, S.S. & Karr, A. Deming, data and observational studies. A process out of control and needing fixing. Significance 116–120 (2011).

[49] Schneeweiss, S. Learning from big health care data. N. Engl. J. Med. 370:2161–2163 (2014). 24897079 10.1056/NEJMp1401111

[50] Psaty, B.M. & Breckenridge, A.M. Mini‐sentinel and regulatory science‐big data rendered fit and functional. N. Engl. J. Med. 370:2165–2167 (2014). 24897081 10.1056/NEJMp1401664

[51] Schneeweiss, S. , Gagne, J.J. , Glynn, R.J. , Ruhl, M. & Rassen. J.A. Assessing the comparative effectiveness of newly marketed medications: methodological challenges and implications for drug development. Clin. Pharmacol. Ther. 90:777–790 (2011). 22048230 10.1038/clpt.2011.235

[52] <http://www.mini‐sentinel.org/work_products/Statistical_Methods/Mini‐Sentinel_Methods_Taxonomy‐Year‐2‐Report.pdf>.

[53] Fleurence, R.L. *et al* Launching PCORnet, a national patient‐centered clinical research network. J. Am. Med. Inform. Assoc. 21:578–582 (2014). 24821743 10.1136/amiajnl-2014-002747 PMC4078292

[54] Arlett, P. *et al* The European Medicines Agency's evidence‐based management of the benefit to risk of medicines. Pharmacoepidemiol. Drug Saf. (2014); e‐pub ahead of print.

[55] <http://www.pmda.go.jp/english/service/mihari_project.html>.

[56] Sutter, S. Safety surveillance using real‐time medical records starts with anticoagulant pilot. The Pink Sheet June 2, 2014, p 10–11.

[57] ASCO Completes Prototype for CancerLinQ, Marking First Demonstration of a “Learning Health System” to Transform Cancer Care. <http://www.asco.org/press‐center/asco‐completes‐prototype‐cancerlinq%E2%84%A2‐marking‐first‐demonstration‐%E2%80%9Clearning‐health>. Accessed 7 July 2014.

[58] FDA . The voice of the patient: a series of reports from FDA's Patient‐Focused Drug Development Initiative. <http://www.fda.gov/ForIndustry/UserFees/PrescriptionDrugUserFee/ucm368342.htm> (2014). Accessed 31 July 2014.

[59] EMA . Patients and consumers. <http://www.ema.europa.eu/ema/index.jsp?curl=pages/partners_and_networks/general/general_content_000317.jsp&mid=WC0b01ac058003500c> (2014). Accessed 31 July 2014.

[60] Genetic Alliance UK . New medicines for serious conditions: weighing the risks and benefits. The verdict of a jury of patients. <http://www.geneticalliance.org.uk/docs/citizens‐jury‐report.pdf>. Accessed 10 July 2014.

[61] Beyer, A. , Fasolo, B. , Hillege, H. , de Graeff, P. & Eichler, H.G. Value and utilities among European patients: testing a decision‐analytic tool for measuring patient preferences. Med. Decision Making. Submitted 13 June 2014.

[62] The EU Pharmacovigilance system . <http://ec.europa.eu/health/human‐use/pharmacovigilance/index_en.htm>. Accessed 3 July 2014.

[63] Dyer, O. Canadian health ministry is given new powers to tighten drug safety. BMJ. 348:g4231 (2014). 24962373 10.1136/bmj.g4231

[64] New Governmental Regulatory System for Stem Cell‐Based Therapies in Japan . Therapeutic Innovation & Regulatory Science March 28, 2014. 10.1177/2168479014526877 30227468

[65] <http://www.lakemedelsverket.se/english/All‐news/NYHETER‐2010/The‐MPA‐investigates‐reports‐of‐narcolepsy‐in‐patients‐vaccinated‐with‐Pandemrix/>.

[66] Vermeer, N.S. *et al* Risk management plans as tool for proactive pharmacovigilance: a cohort study of newly approved drugs in Europe. Clin. Pharmacol. Ther.; e‐pub ahead of print. 10.1038/clpt.2014.184 25222619

[67] IMI GetReal project website. <http://www.imi‐getreal.eu/>. Accessed 3 July 2014.

[68] Pricewaterhousecoopers report: Pharma 2020: The vision. Which path will you take? (2007).

[69] FDA: Structured approach to benefit‐risk assessment in drug regulatory decision‐making. Draft PDUFA V Implementation Plan. February 2013. Fiscal Years 2013‐2017. <http://www.fda.gov/downloads/ForIndustry/UserFees/PrescriptionDrugUserFee/UCM329758.pdf> (2013). Accessed 3 July 2014.

[70] EMA Benefit‐risk Methodology Project. Work package 2 report: Applicability of current tools and processes for regulatory benefit‐risk assessment. <http://www.ema.europa.eu/docs/en_GB/document_library/Report/2010/10/WC500097750.pdf> (2010). Accessed 3 July 2014.

[71] High tech town hall charts path to reform drug development, spur innovation. <http://themorningconsult.com/2014/09/columns‐high‐tech‐town‐hall‐charts‐path‐to‐reform‐drug‐development‐spur‐innovation/>.

[72] Adaptive Licensing Pilot Project. <http://www.ema.europa.eu/ema/index.jsp?curl=pages/regulation/general/general_content_000601.jsp&mid=WC0b01ac05807d58ce>.

[73] Schlich, T. Railways, industry, and surgery‐the introduction of risk management. N. Engl. J. Med. 369:1978–1979 (2013). 24256376 10.1056/NEJMp1309194

