# Supplementary material for: From adaptive licensing to adaptive pathways: Delivering a flexible life‐span approach to bring new drugs to patients
Source: Clin Pharmacol Ther. 2015 Feb 4;97(3):234–46. doi: 10.1002/cpt.59 (PMC6706805; doi:10.1002/cpt.59)
Supplement: Supplementary file 1 — Supporting Information [file CPT-97-234-s001.doc]

**Supplementary information**

Manganiello, M, Anderson, M. Back to Basics. HIV/AIDS advocacy as a model for catalyzing change. <http://forces4quality.org/node/6968> (2011) - HCM Strategists website accessed 3 July 2014

Collier, AK. Penny wise: tiers of pain—is your medication safe from specialty‐tier pricing? Neurology Now June/July 2011 - Volume 7 - Issue 3 - p 42–43

Addressing the Challenges of Serogroup B Meningococcal Disease Outbreaks on Campuses: A report by the National Foundation for Infectious Diseases, May 2014; <http://www.nfid.org/meningococcal-b>

Ollendorf D. If Everyone hates the FDA approval process, let's fix it. http://blogs.hbr.org/2013/10/if-everyone-hates-the-fda-approval-process/ (2013) – HBR Blog Network website accessed 3 July 2014

Strength Happens Together: PPMD Submits FDA Draft Guidance on Duchenne. <http://community.parentprojectmd.org/profiles/blogs/strength-happens-together> (2014)- Parent Project Muscular Dystrophy website accessed 7 July 2014

O’Connor A. Comments to EMEA. <http://www.emsp.org/ms-related-publications/137-allens-oconnors-comments-to-emea-european-agency-for-the-evaluation-of-medicinal-productsaugust-2005> (2005) - EMSP website accessed 3 July 2014

European Brain Council – extract from their manifesto for the 2014 EU elections: [http://www.europeanbraincouncil.org/EUElectionsManifesto/index.asp#4](http://www.europeanbraincouncil.org/EUElectionsManifesto/index.asp#4). EBC website – accessed 3 July 2014.

Mayer M. Listen to all the voices: an advocate's perspective on early access to investigational therapies. Clin Trials. 2006;3(2):149-53.

Flynn TN, Huynh E, Peters TJ, Al-Janabi H, Clemens S, Moody A, Coast J. Scoring the icecap-a capability instrument. Estimation of a UK general population tariff. Health Econ. 2013 Nov 20.

Baghdadi R, Laffler MJ. The Next Phase In Oncology: FDA’s Pazdur Has New Vision For Drug Development. Pink Sheet 2013 Nov 11: 10-12

Jaffe ES, Harris NL, Stein H, Isaacson PG. Classification of lymphoid neoplasms: the microscope as a tool for disease discovery. Blood 2008, 112(12), 4384-4399. <http://bloodjournal.hematologylibrary.org/content/112/12/4384?sso-checked=1>. Blood website - accessed 10 July 2014

Berndt ER, Trusheim M. The Segmentation of Therapeutic Populations in Oncology (2012). Health Management, Policy and Innovation, 1 (1): 19-34, 2012

Jack CR Jr et al. Alzheimer's Disease Neuroimaging Initiative. Serial PIB and MRI in normal, mild cognitive impairment and Alzheimer's disease: implications for sequence of pathological events in Alzheimer's disease. Brain. 2009 May;132(Pt 5):1355-65.

Limb M. Drugs for dementia should receive accelerated approval, says world envoy. BMJ 2014 Jun 20;348:g4166.

Staton T. Pricey hep C drugs, beware: Express Scripts plans a showdown. <http://www.fiercepharma.com/story/pricey-hep-c-drugs-beware-express-scripts-plans-showdown/2013-12-11> (2013) FiercePharma website – accessed 3 July 2014

McCallister E. Out in the cold. BioCentury. The Bernstein Report on BioBusiness Vol 21 (47): A1-A4 (Dec 2013)

Laupacis A, Sackett DL, Roberts RS. "An assessment of clinically useful measures of the consequences of treatment." New England Journal of Medicine 1988;318:1728-33

Zerhouni EA. Turning the Titanic. Sci Transl Med. 2014 Jan 29;6(221):221ed2.

Trusheim MR, Berndt ER. Economic challenges and possible policy actions to advance stratified medicine. Personalized Medicine (2012) 9(4):413-427

Koelsch C, Przewrocka J et al. Towards a balanced value Business… Pharmacogenomics 2013

Kunst M, Natanek R, Plantevin L, Eliades G. A new pharma launch paradigm: From one size fits all to a tailored product approach. <http://www.bain.com/Images/BAIN_BRIEF_A_new_pharma_launch_paradigm.pdf> Bain website - Accessed 4 July 2014

Green Park Collaborative: http://www.cmtpnet.org/resource-center/category/green-park-collaborative/].

Forda SR, Bergström R, Chlebus M, Barker R, Andersen PH. Priorities for improving drug research, development and regulation. Nat Rev Drug Discov. 2013 Apr;12(4):247-8.

Schulthess, D, Chlebus, M, Bergstrom, R and Van Bealen, K. Medicines adaptive pathways t patients (MAPPs): using regulatory innovation to defeat Eroom’s law. Chin Clin Oncol 2014, 3: 21

Svensk Reumatologis Kvalitetsregister. <http://srq.nu/> website accessed 9 July 2014

Barker AD, Sigman CC, Kelloff GJ, Hylton NM, Berry DA, Esserman LJ. I-SPY 2: an adaptive breast cancer trial design in the setting of neoadjuvant chemotherapy. Clinical pharmacology and therapeutics. 2009;86(1):97-100

Avorn J. In Defence of Pharmacoepidemiology – Embracing the Yin and Yang of Drug Research. N Engl J Med. 2007 Nov 29;357(22):2219-21

Rassen JA, Schneeweiss S. Using high-dimensional propensity scores to automate confounding control in a distributed medical product safety surveillance system. Pharmacoepidemiol Drug Saf. 2012 Jan;21 Suppl 1:41-9.

Obenchain RL, Young SS. Advancing statistical thinking in observational health care research. Journal of Statistical Theory and Practice, 7:456-459, 2013.

Curtis LH, Brown J, Platt R. Four health data networks illustrate the potential for a shared national multipurpose big-data network. Health Aff (Millwood). 2014 Jul;33(7):1178-86

Curtis LH, Weiner MG, Boudreau DM, Cooper WO, Daniel GW, Nair VP, Raebel MA, Beaulieu NU, Rosofsky R, Woodworth TS, Brown JS. Design considerations, architecture, and use of the Mini-Sentinel distributed data system. Pharmacoepidemiol Drug Saf. 2012 Jan;21 Suppl 123-31

FDA. Bringing Your Voice to Drug and Device Approval and Safety. <http://patientnetwork.fda.gov/get-involved/become-patient-representative> (2014) FDA website - accessed 31 July 2014

<http://www.ema.europa.eu/docs/en_GB/document_library/Other/2014/03/WC500163409.pdf>

<http://www.fda.gov/ForIndustry/UserFees/PrescriptionDrugUserFee/ucm326192.htm>

http://www.cadth.ca/en/products/cdr/patient-group-input

<http://www.pcodr.ca/wcpc/portal/Home/SubmitAndContribute?_afrLoop=315417704453000&_afrWindowMode=0&_adf.ctrl-state=vawx2j6tf_91>

Axel C Mühlbacher, Susanne Bethge, Susanne EblePatient preferences for Integrated Care Networks in Germany: A Discrete-Choice Experiment (DCE). Int J Integr Care 2013; Annual Conf Suppl; URN:NBN:NL:UI:10-1-114674

Institute of Medicine. The future of drug safety: promoting and protecting the health of the public. Washington, DC: National Academies Press, 2006.

http://www.pmda.go.jp/english/service/rmp.html
